# Brentuximab-Induced Acute Interstitial Nephritis: A Case Report

**DOI:** 10.1177/20543581241300766

**Published:** 2024-11-25

**Authors:** Matthew Patterson, Pouneh Dokouhaki, Chance S. Dumaine, Rebecca MacKay, Davina J. Tai

**Affiliations:** 1 Division of Nephrology, Department of Medicine, University of Saskatchewan, Saskatoon, Canada; 2 Department of Pathology and Lab Medicine, University of Saskatchewan, Saskatoon, Canada; 3 Division of Hematologic Oncology, Department of Medicine, University of Saskatchewan, Saskatoon, Canada

**Keywords:** interstitial nephritis, AKI (acute kidney injury), brentuximab

## Abstract

Brentuximab vedotin is a combination monoclonal antibody to anti-CD30 conjugated to the anti-tubulin agent monomethyl auristatin E. It is approved for the treatment of mycosis fungoides, Hodgkin’s lymphoma, and systemic anaplastic large cell lymphoma. Brentuximab has been associated with a number of potential adverse reactions; however, reports of renal complications are rare. A 73-year-old male with mycosis fungoides was admitted to hospital with acute kidney injury following his third cycle of brentuximab. The patient’s serum creatinine (SCr) was 122 µmol/L with an estimated glomerular filtration rate (eGFR) of 58 mL/min/1.73 m^2^ at baseline. Following brentuximab, his SCr peaked at 1073 µmol/L over a 4-week period. Acute interstitial nephritis (AIN) was diagnosed after other causes of acute kidney injury were ruled out and subsequently confirmed on kidney biopsy. The patient was started on prednisone 50 mg daily. This was continued for 3 weeks, followed by a 5-week taper. The patient’s SCr decreased to 156 µmol/L by completion of the prednisone taper. He was not rechallenged with brentuximab. A kidney biopsy confirmed AIN in keeping with injury from an immune checkpoint inhibitor (ICI). However, brentuximab is not an ICI. The AIN from ICIs typically has tubulointerstitial inflammatory infiltrate comprised of T lymphocytes such as the case presented here. Therefore, this represents both a novel histopathologic finding in AIN from a non-ICI medication and a rare complication of brentuximab, previously only presented in abstract form.

## Résumé

Le brentuximab vedotin est un médicament immunoconjugué, composé d’un anticorps monoclonal anti-CD30 et de l’agent anti-tubuline monométhyl auristatine E. Il est approuvé pour le traitement du mycosis fongoïde, du lymphome de Hodgkin et du lymphome anaplasique systémique à grandes cellules. Le brentuximab a été associé à un certain nombre d’effets indésirables potentiels, mais les cas de complications rénales sont rares.

Un homme de 73 ans atteint de mycosis fongoïde a été admis à l’hôpital pour insuffisance rénale aiguë après son troisième cycle de brentuximab. Les mesures initiales de créatinine sérique (CrS) et le débit de filtration glomérulaire estimé (DFGe) étaient respectivement de 122 µmol/L et de 58 mL/min/1.73 m^2^. Après le traitement par brentuximab, son niveau de CrS a atteint un pic à 1 073 µmol/L sur une période de quatre semaines.

Une néphrite interstitielle aiguë (NIA) a été diagnostiquée après que l’on eut écarté d’autres causes d’insuffisance rénale aiguë, puis confirmée par biopsie rénale. Le patient a entrepris un traitement par prednisone 50 mg/jour qui s’est poursuivi pendant trois semaines, avant une réduction progressive sur cinq semaines.

Le niveau de CrS du patient est passé à 156 µmol/L après le sevrage de la prednisone. Le brentuximab n’a pas été réintroduit.

Une biopsie rénale a confirmé que la NIA correspondait aux lésions causées par un inhibiteur du point de contrôle immunitaire (IPCI). Or, le brentuximab n’est pas un IPCI. Les NIA causées par les IPCI présentent typiquement un infiltrat inflammatoire tubulointerstitiel composé de lymphocytes T, comme dans le cas de ce patient. Ce cas représente donc à la fois une nouvelle découverte histopathologique pour les NIA causées par un médicament non IPCI et une complication rare du brentuximab qui était jusqu’à maintenant présentée uniquement sous forme d’abrégés.

## Introduction

Acute interstitial nephritis (AIN) is a common cause of acute kidney injury (AKI), characterized by inflammatory infiltrate against either endogenous or exogenous nephritogenic antigens.^1^ Although there are many possible etiologies of AIN, drug-induced AIN remains the most common, believed to represent 60% to 70% of cases.^2^

Brentuximab vedotin (hereafter referred to as brentuximab) is an antibody-drug conjugate of anti-CD30 monoclonal antibody combined with the anti-tubulin agent monomethyl auristatin E. It is approved for treatment of mycosis fungoides, Hodgkin’s lymphoma, and systemic anaplastic large cell lymphoma.^3,4^ Brentuximab has been associated with several potential adverse reactions; however, reports of renal complications have been rare.^5^ Here, we present a case of brentuximab-induced AIN.

## Case Report

### Presenting Concerns

A 73-year-old male with a past medical history of hypertension, dyslipidemia, and benign prostatic hypertrophy was initiated on brentuximab after a new diagnosis of mycosis fungoides. He had stage G3aA1 chronic kidney disease (CKD) from hypertension with a baseline SCr of 130 µmol/L and estimated glomerular filtration rate (eGFR) of 58 mL/min/1.73 m^2^, as determined by the 2009 CKD-Epidemiology Collaboration (CKD-EPI) equation. His kidney function initially remained stable for the first 2 months of brentuximab. However, routine bloodwork completed between his third and fourth 3-week cycles of brentuximab revealed an SCr of 647 µmol/L. The patient was admitted to hospital 2 days later for further investigations and management of his AKI.

Upon presentation to hospital, the patient stated he had been feeling well with no symptoms to suggest intravascular volume depletion, urinary tract obstruction, or systemic vasculitis. He denied any rashes, arthralgias, or malaise. The only notable symptom was an increase in nocturia over the preceding few weeks. His pre-admission medications included rosuvastatin, telmisartan, and pantoprazole. He had been on pantoprazole 40 mg po daily for many years. The patient had also taken 2 tablets of regular strength naproxen twice daily for 2 days the week prior to his hospital admission.

### Clinical Findings

His admission vital signs included a blood pressure of 161/88 mm Hg, heart rate of 93 beats per minute, oxygen saturation of 99% on room air, and axial temperature of 37.5°C. The remainder of his physical examination was unremarkable.

Admission laboratory work revealed a further increase in SCr to 906 µmol/L, blood urea nitrogen (BUN) of 23.8 mmol/L, serum albumin of 31 g/L, and urine albumin to creatinine ratio of 15.82 mg/mmol. Serum eosinophils were normal at 0.02 × 10^9^/L. Urinalysis was positive for 2+ leukocyte esterase, 2+ protein, 1+ blood, and negative for the presence of nitrites. Urine microscopy showed 20 to 50 white blood cells per high powered field, and 3 to 5 red blood cells per high powered field. Urine culture was negative. Hepatitis B, hepatitis C, and human immunodeficiency virus serologies were negative. Antineutrophil cytoplasmic antibody, antinuclear antibody, and anti-glomerular basement membrane antibody tests were negative; serum protein electrophoresis results and complement components C3 and C4 concentrations were normal. A renal ultrasound indicated normal kidney size, echogenicity, and no evidence of hydronephrosis.

### Diagnostic Focus and Assessment

**Figure 1.**
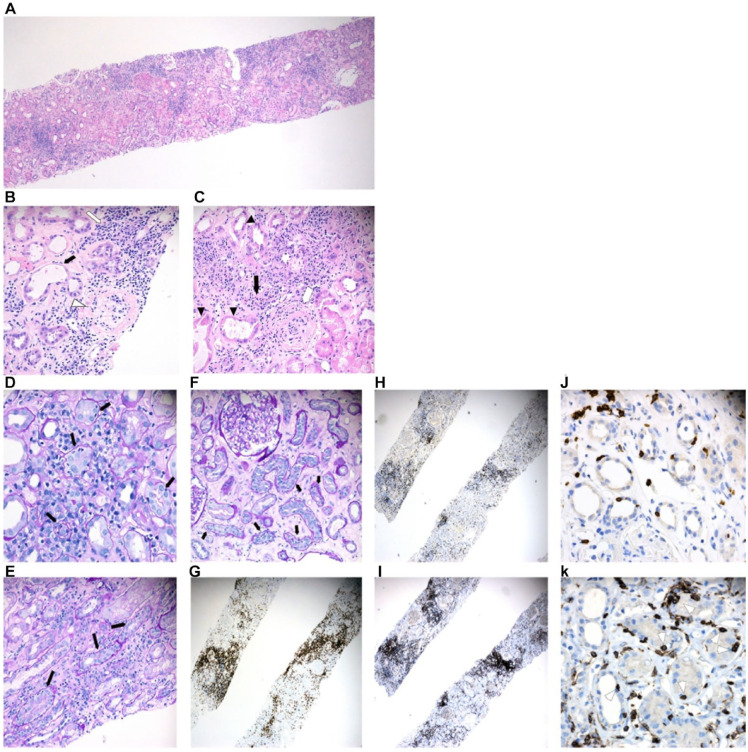
(A) Kidney core biopsy, H&E stain, 40×. (B) Interstitial and glomerular inflammation (arrowhead) consisting of lymphocytes, histiocytes, and plasma cells (white arrow); tubular injury seen as epithelial cell flattening and loss of brush border (black arrow); H&E stain, 200×. (C) Focal aggregates of eosinophils (black arrow) adjacent to acute tubular injury (arrowheads) and an invaded glomerulus (white arrow); H&E stain, 200×. Panels (D), (E), and (F) demonstrate numerous areas of mild-to-moderate lymphocytic tubulitis (black arrows); PAS stain 200×. (G) Infiltrating lymphocytes are mostly of T-cell lineage and are CD3-positive. (H) and (I) show immunohistochemical staining of CD8 and CD4 T cells, respectively, with the majority being CD4-positive helper T cells. (J) and (K) show some CD8+ cytotoxic T cells invading the tubules, but the dominant population of invading lymphocytes are CD4+ helper T cells (arrow heads).

A diagnosis of AIN was strongly suspected. The patient’s SCr peaked at 1073 µmol/L on post admission day 2. A kidney biopsy was performed 3 days later. This revealed diffuse and multifocal dense interstitial inflammation composed of predominantly small lymphocytes with admixed plasma cells and rare clusters of eosinophils (Figure 1). Acute tubular injury with many foci of lymphocytic tubulitis causing severe epithelial cell damage was easily appreciated. Remnants of glomeruli destroyed by the invading active lymphocytes were noted. Characterization of lymphocytic infiltrate by immunophenotyping showed that the dominant population was CD3+ T lymphocytes. Although both helper T cells (CD4+) and cytotoxic T cells (CD8+) were present among the infiltrates, the majority of infiltrating T lymphocytes, particularly those invading and destroying the tubules, were of CD4+ phenotype. No granulomatous inflammation was seen. Immunofluorescent studies and electron microscopy showed unremarkable glomerular structure with no notable immune deposits in the glomeruli or extraglomerular locations.

### Therapeutic Focus and Assessment

The patient was started on oral prednisone 50 mg daily beginning post admission day 2, given the severity of his AKI. His pantoprazole, telmisartan, and brentuximab were discontinued on admission.

### Follow-up and Outcomes

**Figure 2.**
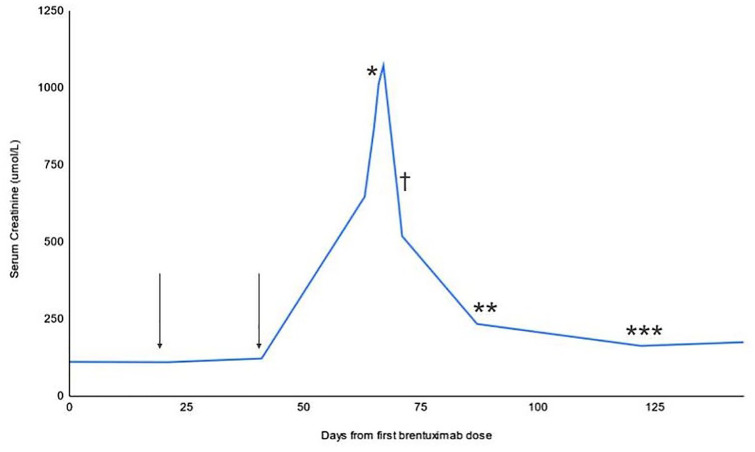
Timeline of events expressed as serum creatinine from the day of first brentuximab exposure. Vertical arrows represent the second and third brentuximab doses; * represents the initiation of prednisone; ** represents the initiation of prednisone taper; *** represents prednisone completion, and † represents the date of kidney biopsy.

The patient was discharged after 6 days in hospital with an 8-week prednisone course (50 mg daily for 3 weeks followed by a taper of 10 mg weekly until at 10 mg daily, then 5 mg daily for one week). Upon discharge, his SCr had decreased to 519 µmol/L. Post discharge, his SCr continued to improve over 7 weeks, decreasing by approximately 40 µmol/L per week until reaching a nadir of 156 µmol/L (Figure 2). The patient’s kidney function remained stable off prednisone; his SCr was 164 µmol/L and eGFR was 41 mL/min/1.73 m^2^ 8 months following his AKI diagnosis. There were no adverse events related to his prednisone therapy.

## Discussion

We report a case of AIN due to brentuximab. Classically, drug-induced AIN has been associated with antibiotics, non–steroidal anti-inflammatory drugs (NSAIDs), proton pump inhibitors (PPIs), and more recently, ICIs.^2,6^ Typical adverse events from brentuximab include neuropathy, neutropenia, diarrhea, vomiting, alopecia, pyrexia, fatigue, and anemia.^5^ Despite well-known potential adverse effects, renal complications associated with brentuximab have been rarely reported. Only 2 prior case reports of AIN following brentuximab administration have been presented in abstract form.^7,8^ The first case described a 50-year-old female with anaplastic high-grade Hodgkin’s lymphoma and karyomegalic interstitial nephritis requiring dialysis.^7^ She was not treated with glucocorticoids and remained dialysis dependent. The second case involved a 64-year-old female with cutaneous T-cell Lymphoma whose AIN resolved after treatment with prednisone.^8^ Differences exist between our case and the 2 previously published reports of brentuximab-induced AIN. First, our case did not show evidence of karyomegalic AIN. Second, we provide greater detail on the type of T lymphocytes present and provide a possible mechanism for brentuximab-induced AIN as described below.

Although our patient had other potential causes of AIN (2 doses of naproxen prior to his presentation and 2 years of pantoprazole use), we point to brentuximab as the causative agent. The patient’s kidney function had been stable for years while taking pantoprazole, making it less likely that this was the etiology. The rise in SCr also preceded his NSAID use. In addition, AIN occurred after our patient’s third cycle of brentuximab, similar to the aforementioned case reports in which AIN occurred after 2 cycles.^7,8^

Our patient’s biopsy also demonstrated histopathologic features not typically seen with NSAID or PPI-associated AIN. The tubulointerstitial inflammatory infiltrate was comprised mainly of T lymphocytes that invaded and destroyed the tubules. This is unlike the interstitial inflammation induced by NSAIDs and PPIs in which the infiltrate is commonly eosinophil or plasma cell rich with minimal direct damage to the tubular cells.^2^ Interestingly, the biopsy findings in our case closely resemble the biopsy findings more commonly seen in ICI associated AIN. The AIN from ICIs is caused by invading CD8+ T lymphocytes that have lost tolerance against endogenous antigens.^9^ The difference in our case is that the predominant T lymphocytes were of the CD4+ phenotype. Brentuximab is not an ICI but contains a monoclonal antibody against CD30. The CD30 molecule exerts a regulator function on CD4+ but not on CD8+ T lymphocytes.^10^ It is possible that once this regulatory effect is lifted by the anti-CD30 neutralizing antibody, the unexpected autoreactivity of CD4+ T-cell population is unleashed leading to AKI from AIN.

In conclusion, we report a case of AIN induced by brentuximab. The AIN resolved after brentuximab cessation and a course of corticosteroids. Prescribers should be aware that brentuximab therapy may result in severe AIN and careful monitoring of kidney function is warranted.
